# Overcoming Impediments to the Qualification of Additively Manufactured Polymer Components: The Case of ULTEM

**DOI:** 10.3390/polym18121477

**Published:** 2026-06-12

**Authors:** Colin Marquis, Vanessa Bradshaw, Anushka Sarode, Megan Hong, Lars Glaesner, Ellen Ma, Mark Sorna, Dwayne Arola

**Affiliations:** 1 Department of Materials Science and Engineering, University of Washington, Seattle, WA 98195, USA; cdm17@uw.edu (C.M.); vanebrad@uw.edu (V.B.);; 2 Department of Materials Science and Engineering, Carnegie Mellon, Pittsburg, PA 15213, USA; 3 Naval Undersea Warfare Center, Naval Base, Keyport, WA 98345, USA; mksrna@yahoo.com; 4 Department of Mechanical Engineering, University of Washington, Seattle, WA 98195, USA

**Keywords:** additive manufacturing, fused deposition modeling, ULTEM™ 9085, ULTEM™ 1010, thermoplastics, material qualification

## Abstract

The qualification of additively manufactured (AM) components produced from engineering polymers poses unique challenges, particularly when evaluating mechanical properties according to ASTM D638. The application of high-performance thermoplastics, such as ULTEM™ 9085 and ULTEM™ 1010, frequently relies on manufacturer-provided datasheets for qualification. However, existing datasheets do not provide guidance specific to articles printed in the XY plane, which can be complicated by failures that initiate at microstructural anomalies rather than being driven by intrinsic material behavior. The objective of this study was to investigate the performance and qualification of ULTEM 9085™, examined according to ASTM D638, and pursue improvements through refined print parameters. A significant improvement in strength and conforming failures was achieved with modest adjustments to the print settings. For Type 1 samples printed with ±45° infill, gage section failures improved from only 5% to 100%, while samples with 0/90° infill achieved 80%. Correspondingly, the ultimate tensile strength increased from 49 ± 2 MPa to 61 ± 2 MPa and from 53 ± 3 MPa to 63 ± 6 MPa, respectively. These results underscore the critical role of process parameters, including contour overlap, in qualifying polymer AM materials, and their contribution to the performance and reliability of printed components.

## 1. Introduction

The Fused Deposition Modeling^®^ (FDM) process, enabling the layer-by-layer manufacturing of polymer components directly from a computer-aided design (CAD) model, was introduced by Stratasys in the late 1980s [1]. Now part of a larger family of additive manufacturing (AM) technologies, FDM offers greater design flexibility and generates less waste material than traditional subtractive manufacturing methods [2,3]. It enables the manufacturing of parts that would be impossible to produce with traditional techniques [4]. Fused filament fabrication (FFF), its open-source counterpart, and FDM are now widely used to produce prototypes and low-volume production parts across many industries.

The FDM process involves extruding a polymer feedstock through a heated nozzle and depositing the melted material onto a print bed or a prior layer in a sequential manner to build the component to net shape [5]. The mechanical performance of components printed via FDM is inherently anisotropic, with the direction transverse to the layers representing the weakest overall. In addition to this inherent anisotropy, there is an aspect of anisotropy related to the printed microstructure that is a function of the printing parameters selected for the filament feedstock; the infill orientation is a primary contributor [6]. The qualification of mechanical behavior is key for applications in many industries, and suppliers typically provide guidelines that define the printing parameters necessary to achieve the reported values outlined in the manufacturer’s datasheet. Herein lies the concern.

The tensile properties of the thermoplastics used in FDM are typically evaluated according to ASTM D638, which recommends specific dog-bone coupon geometries based on the material of interest. The standard, which was developed for polymers independent of the method of manufacturing, does not specify parameters related to FDM. For AM materials, the design or manufacturing engineer must rely on the material datasheets and qualification processes outlined by material suppliers [7]. In addition, mechanical properties should be reported in alignment with ASTM F2971, the standard practice for reporting data for test samples prepared by AM [8]. Indeed, Stratasys provides material datasheets (MDSs) with properties reported for evaluations that conform to the ASTM D638 standard. The coupons are printed following the recommended settings and seam control adjustments outlined by Stratasys for the material [9]. Importantly, there are often microstructural features present in Type 1 coupons of ASTM D638 that have been printed using these recommended settings that can adversely affect the location of failure [10,11]. These suboptimal failure locations can adversely influence the mechanical properties recorded [12,13]. Furthermore, the MDS only includes results for coupons printed in the XZ (on-edge) and ZX (Upright) orientations, which correspond to the strongest and weakest loading configurations, respectively [8]. This approach is used by Stratasys for the MDS of their high-performance thermoplastic materials, such as ULTEM™ 9085 and ULTEM™ 1010 [9]. Values are not provided for the XY (flat) printing orientation, which is often preferred by AM users and would be the most common for maximizing surface contact with the build plate. Nonconforming failures often occur with XY orientation prints when evaluated by traditional standards like ASTM D638. The MDSs, which clearly define the minimum and maximum strengths when fabricated using AM, appear to omit these values due to either low strengths attributable to the nonconformities or other complications encountered in the evaluation process.

ULTEM™ 9085 and 1010 are proprietary Polyetherimide (PEI) polymer blends developed by Sabic Innovative Plastics and are printed with Stratasys FDM systems to produce high-strength polymer parts [14,15]. ULTEM™ 9085 is a mixture of PEI and Polycarbonate (PC), while ULTEM™ 1010 is pure PEI. These high-performance thermoplastic polymers are regarded for their high melting point, chemical stability, and exceptional strength [16,17]. They are common choices for operators seeking a high-performance engineering thermoplastic [18]. Owing to the shear-thinning effects of the PC component, ULTEM™ 9085 is more often used in commercial part production due to its enhanced printability [19]. Additionally, the chemical stability of PEI makes it an excellent candidate for applications where degradation is a concern due to environmental exposures [16]. There is particular interest in the adoption of these materials for applications in marine structures and unmanned underwater vehicles (UUVs) [17], which is an ideal complement to the response readiness provided by AM processes [20].

For performance-critical applications where reliability is key, there is some concern in the adoption of these AM polymers, namely, the wide range of mechanical properties reported in the literature compared to the narrow range outlined in the MDS. Padovano et al. provide possibly the most comprehensive comparison of properties for ULTEM™ 9085 evaluated according to ASTM D638 with Type 1 coupons printed using ±45° infill. They identified that the void content increases proportionally with the percentage raster area per layer and decreases with the percentage contour area [18]. Zaldivar et al. reported that stress concentrations are present in the printed microstructure at the transition from the grip to the gage section [21]. Ahn et al. addresses anisotropy in AM prints as a function of build parameters in printing ABS, which translates to other high-performance polymers such as ULTEM™ [22]. Similar studies have also been conducted for ULTEM™ 9085 and 1010 regarding all three printing orientations (XZ, ZX, and XY), exhibiting varying levels of agreement with the MDS specifications [10,18,21,23,24]. For ULTEM™ 9085, the ultimate tensile strengths (UTSs) reported in the literature for the XY printed orientation range between roughly 45 and 65 MPa [10,18,21,23,24]. It is hypothesized that this variability could be associated with the coupon geometry of the standard and the mesostructure achieved by the FDM process when printed with the recommended parameters [18,25,26]. Appropriate adjustment in parameters and/or geometry could improve strength, narrow variability, and increase reliability [27,28].

The present investigation aims to qualify these ULTEM™ materials, first, by using the recommended printing parameters for various coupon geometries and then by implementing parameter changes to better understand how AM materials can be more effectively qualified. While this work investigates ULTEM™ 9085 and ULTEM™ 1010 specifically, the methodology serves as a framework for addressing additional material systems and AM methods tested in accordance with ASTM D638. Modifications are proposed to address defects in the mesostructure of AM ASTM D638 Type 1 and Type 4 coupons printed with both ±45° and 0/90° infills. The work demonstrates that with proper modifications, greater consistency in the properties of high-performance AM materials is achievable, improving the qualification process.

## 2. Materials and Methods

**Figure 1:**
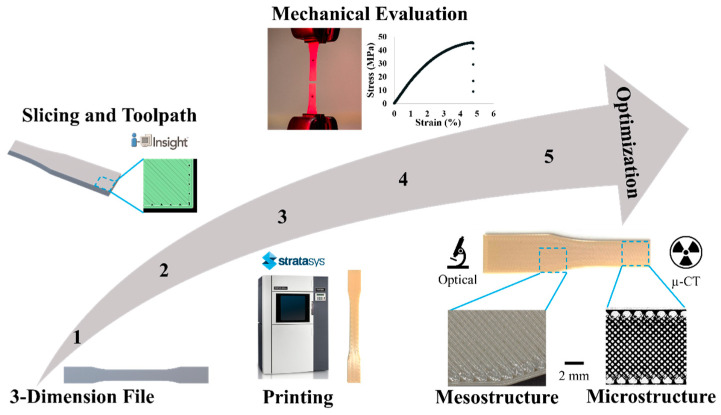
Methodology overview for design, evaluation and optimization of ASTM D638 AM samples.

The process followed to prepare, manufacture, and characterize the polymer components is shown in Figure 1. An iterative design cycle was implemented to improve the mechanical performance of samples fabricated, yield increasingly desirable mesostructure and microstructure, and increase the frequency of conforming failures.

### 2.1. Printing

Tensile coupons of ULTEM™ 9085 and ULTEM™ 1010 were produced with appropriate support material (SUP9000B) by The Naval Undersea Warfare Center (NUWC, NUWC, Keyport, WA, USA) using a commercial printer (Fortus 900mc, Stratasys, Eden Prairie, MN, USA). The ULTEM™ 9085 tensile coupons were printed using T16A tips for the model material, whereas the ULTEM™ 1010 tensile coupons were printed using a T14A tip. The breakaway supports were printed using T16 tips for both materials. The traditional approach was used for fabrication, including the computer-aided design (CAD) of the coupon geometry and slicing for layerwise printing by FDM, which was performed using Insight software (Ver 16.38, Control Center 16.3, Stratasys, Eden Prairie, MN, USA). Slicing followed the Stratasys recommended print settings, including a layer thickness of 0.254 mm and seam control. All coupons were printed with a total thickness of 1.524 mm.

Additional coupons with modified infill were produced from the ULTEM™ 9085 and 1010 materials and printed with a Stratasys printer (Fortus 400mc, Eden Prairie, MN, USA). ULTEM™ 9085 was printed with a T16 tip for the model and support material, while ULTEM™ 1010 was printed with a T14 tip for the model and a T16 tip for the support. CAD models were sliced using Insight software (Ver 18.6, and Control Center 18.6, Stratasys, Eden Prairie, MN, USA).

**Figure 2:**
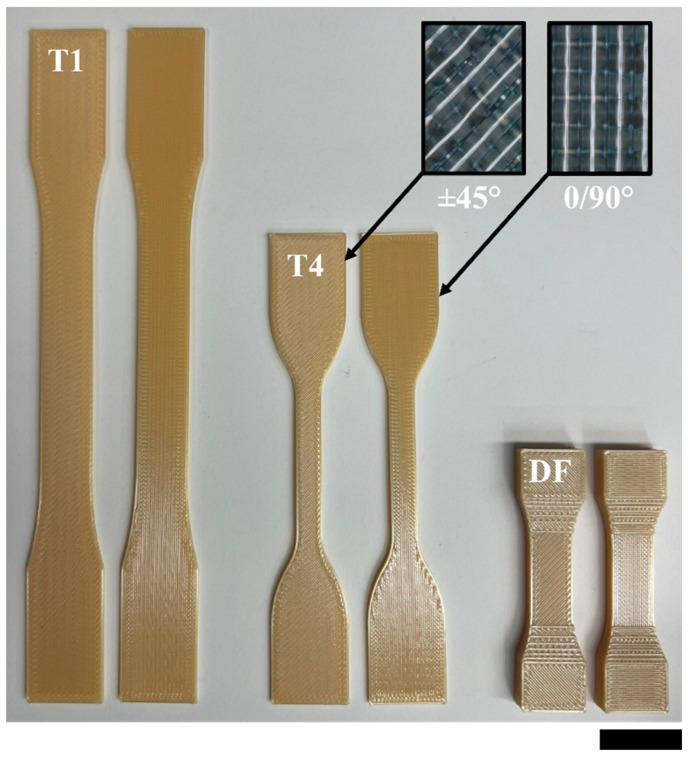
ASTM D638 Type 1 (T1) and Type 4 (T4) sample geometries as well as the proposed Dual Flange (DF) sample geometry. The scale bar is 20 mm.

Tensile samples were printed with three different geometries, including Type 1 and Type 4 of ASTM D638-22, as well as the newly proposed Dual Flange coupon geometry [10]. Although ASTM D638-22 is not explicitly written for testing 3D-printed polymers, there is no existing standard for the evaluation of coupons produced by AM. As a result, this standard is widely accepted across the literature [10,14,15,16,17,18,29,30]. Coupons were printed with two infill types, including ±45° and 0/90° raster patterns. The Dual Flange sample features a reduction in the material cross-section in both the x-axis and the z-axis, as shown in Figure 2. This sample is proposed to minimize failures outside the gage section during tensile testing associated with traditional dog-bone or dumbbell-shaped geometries used in AM [31]. A minimum of 30 samples were printed on the Fortus 900mc with each infill pattern and sample geometry defined by the standard, resulting in a minimum of 180 samples prepared with the ULTEM™ 9085 and ULTEM™ 1010 materials. Additional samples were printed on the 900mc and evaluated as needed based on sample failures.

**Figure 3:**
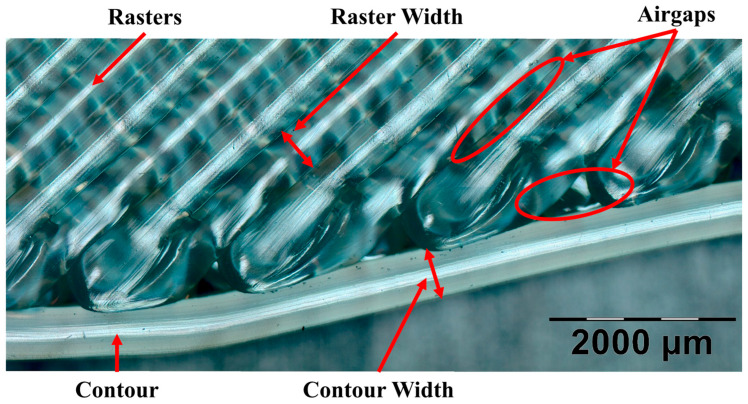
The printed mesostructure for a Type 1 sample within the transition region. The details of the printed features and interface defects (airgaps) are highlighted.

Several parameters can be controlled and modified in FDM, including the raster width, contour width, and the “airgap,” as noted in Figure 3. Stratasys slicing software (Insight) uses the airgap to define the space between adjacent rasters and contours. The default airgap is 0 mm, indicating no overlap of material at contour–contour, contour–raster, or raster–raster interfaces.

### 2.2. Mechanical Testing

The tensile testing of the samples was conducted using a commercial universal testing frame (Model 5585H, Instron Corporation, Norwood, MA, USA), equipped with a 50 kN load cell under displacement control at a stroke rate of 5 mm/min. A video extensometer (AVE 1, Instron Corporation, Norwood, MA, USA) was used to orient the samples in the mechanical wedge grips and to measure strain during the experiment. The instantaneous load and strain were used to prepare stress–strain curves and then determine the elastic modulus, UTS, and strain to failure. Fractographic evaluation was performed to identify samples that failed within the acceptable range as defined by ASTM D638-22 [7]. Samples that failed within the gage section were included for material qualification, whereas failures that did not conform to the standard were evaluated but were not included in the property assessment. A minimum of *N* = 5 samples with acceptable failures are included in the qualification data reported for each sample geometry and infill.

A two-parameter Weibull analysis was performed with the measured strengths according to the following:

(1) $$P(\sigma )\;=\;1-exp\left(-{\left(\frac{\sigma }{\sigma_{0}}\right)}^{m}\right)$$

where *P*(*σ*) represents the probability of failure at the axial stress (*σ*), m is the Weibull modulus, and $\sigma_{0}$ is the characteristic strength. The probability function for failure ($P_{f}$) was defined according to the following estimator function

(2) $$P_{f}=\frac{i-0.5}{N}$$

where *i* represents the *i*th sample in the ranking, and *N* is the total sample count in the group. The Weibull parameters for each sample geometry and infill were compared to assess contributions from the printed quality and specific aspects of the microstructure to the variability in strength.

### 2.3. Microstructure and Surface Analysis

Micro-Computed Tomography (micro-CT) was performed to evaluate the micro- and mesostructures of selected tensile samples using a commercial instrument (NSI X5000 CT, North Star Imaging, Rogers, MN, USA). The scans were conducted with a resolution of 19 μm per pixel. Sequential images were compiled to create a 3D model of the samples to enable the visualization of features of interest and their locations. Porosity was calculated from 3D reconstructions of the voxels through post-processing performed in Dragonfly software version 2024.1 (Comet Technologies, Montréal, QC, Canada).

Contact profilometry was conducted using a MarSurf XR20 to collect information about the roughness average (R_a_), maximum valley depth (R_v_), and reduced valley depth (R_vk_) (MarSurf, Waukesha, WI, USA). Scans were conducted with a traverse length of 5.6 mm, and a cutoff length of λ_c_ = 0.8 mm, within the gage section, following ANSI B46.1, Surface Texture standard (York, NY, USA) [32]. Profilometry enabled the evaluation of the printed coupon surface topography on both the top and build plate surfaces of the printed coupon geometries.

## 3. Results

### 3.1. Surface Morphology

**Figure 4:**
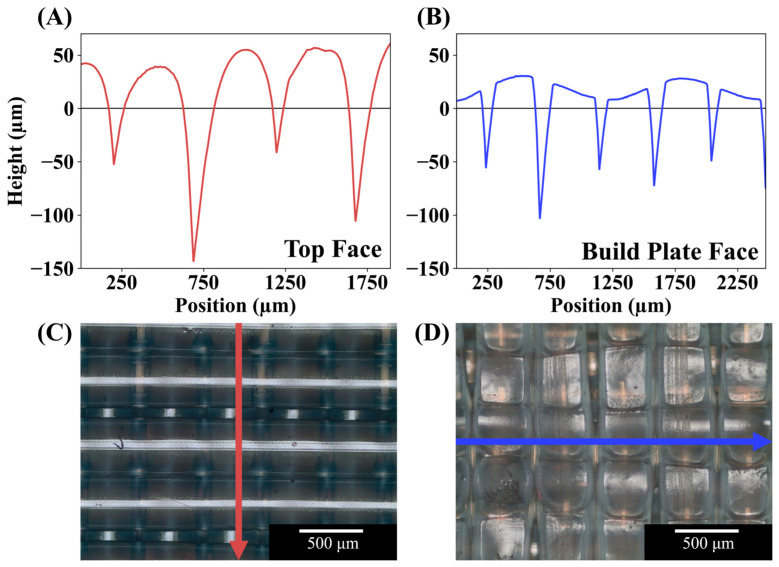
Optical images and corresponding contact profilometry taken for both top (**A**,**C**) and build plate (**B**,**D**) faces of Type 1 ULTEM™ 9085 coupons with arrows indicating scan direction.

Representative surface profiles from the top and build plate sides of representative printed samples are shown in Figure 4A and Figure 4B, respectively. The corresponding optical images of these surfaces are shown in Figure 4C and Figure 4D, respectively. All coupons exhibited an impression of the support material on the build plate face of the coupon. Scans acquired perpendicular to the print direction revealed the uneven spacing of the extruded rasters, with increased separation and depth of valleys between every second contour. This increased spacing resulted in deep valleys of beyond 120 µm in depth on both the top and build plate face, as evident from the R_v_ values listed in Table 1 and Table 2.

**Figure 5:**
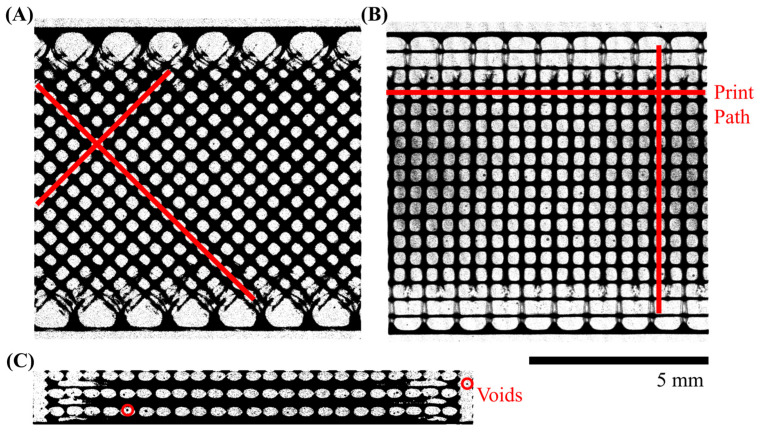
Micro-CT scans of the internal cross-section of two Dual Flange samples viewed through the z-axis, (**A**) ±45°, (**B**) 0/90°, and (**C**) a Type 1 coupon viewed through the x-axis 0/90°. Image stacks show voids (black) projected through all layers of the sample.

Figure 5 shows the internal mesostructures of representative Dual Flange and Type 1 coupons scanned using micro-CT as a projection through all layers. Additional scans were conducted to evaluate the internal mesostructures of Type 1 and Type 4 coupons. Voids were identified throughout the printed contours of both the ULTEM™ 9085 and 1010 materials [33]. The white regions in the CT projection represent a polymer that is continuous across all z-axis slices, corresponding to well-fused contours and overlapping print path intersections. The black regions of the figure indicate the presence of a void in at least one slice within the stack, occurring primarily at contour interfaces and within individual print paths.

Representative samples of each group printed with the default conditions were randomly selected, and the gage section of each was subjected to micro-CT to evaluate porosity. For the samples with a ±45° raster, the Type 1, Type 4, and Dual Flange samples had average porosities of 11.7%, 12.0%, and 12.8%, respectively. For samples with the 0/90° rasters, the average porosities for these three coupons were 5.5%, 15.1%, and 11.2%, respectively. It is expected that ±45° raster samples exhibit greater void content when compared to 0/90° raster samples due to the differences in toolpaths generated to fill the rectangular volume of the gage section, yielding lower contour wall linear density in the ±45° raster samples.

### 3.2. Mechanical Properties

**Figure 6:**
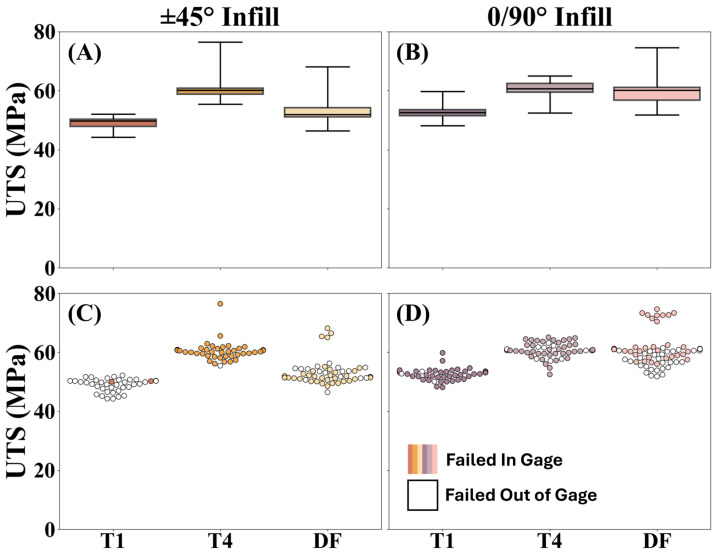
UTS values for ULTEM™ 9085 with ±45° (**A**,**C**) and 0/90° (**B**,**D**) raster patterns showing statistical distribution in strength (box and whisker (**A**,**B**)) and individual sample failure location with strength (swarm (**C**,**D**)).

The results from tensile testing of the ULTEM™ 9085 samples with the three different sample geometries are shown in Figure 6. Specifically, the results for the ±45° and 0/90° infill patterns are shown as box-and-whisker plots in Figure 6A and Figure 6B, respectively, and then as swarm plots in Figure 6C and Figure 6D, respectively, to highlight the span of discrete strength measurements and distinguish responses corresponding to failures within and outside of the gauge region.

For ULTEM™ 9085, the Type 1 ±45° and 0/90° samples exhibit significantly lower UTS than the Type 4 (*p* ≤ 0.0001) and Dual Flange samples (*p* ≤ 0.0001). In addition to the low UTS of the Type 1 samples, those printed with an infill pattern of ±45° have a greater percentage of failures outside the acceptable gage section. In fact, an inadequate number of samples failed within the gage section to report a UTS with confidence for the ±45° infill samples. Specifically, only two failures of 39 tests occurred within the gage section, resulting in a UTS of 50.1 ± 0.1 MPa. A substantially larger number of the Type 4 and Dual Flange samples failed in the acceptable region. The overall UTS for each sample geometry and infill, accounting for and ignoring the location of failure, is presented in Table 3. Comparing samples printed with alternate infill patterns, those with 0/90° infill generally exhibit a higher UTS than those with ±45° infill.

**Figure 7:**
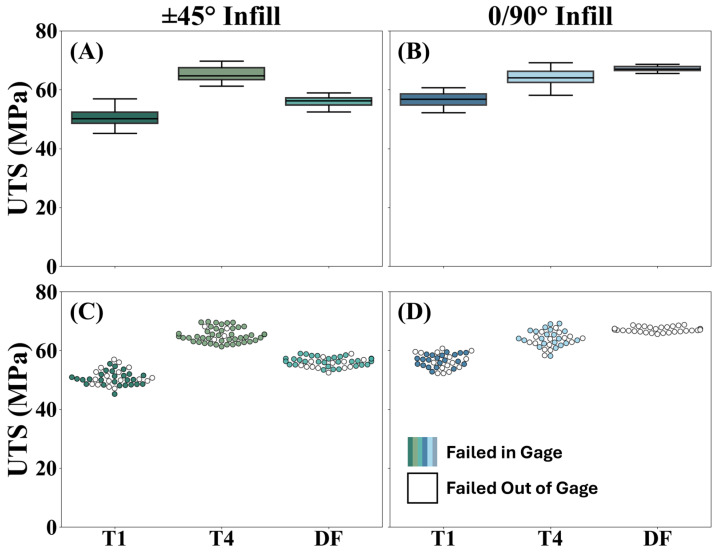
UTS values for ULTEM™ 1010 with ±45° (**A**,**C**) and 0/90° (**B**,**D**) raster patterns, showing statistical distribution in strength (box and whisker (**A**,**B**)) and individual sample failure location with strength (swarm (**C**,**D**)).

The results from the tensile testing of the ULTEM™ 1010 samples with the three different sample geometries are shown in Figure 7 as box-and-whisker plots for the ±45° and 0/90° infill patterns in Figure 7A and Figure 7B, respectively. The corresponding swarm plots are shown in Figure 7C and Figure 7D, respectively. For ULTEM™ 1010, the number of failures that occurred within the gage section is lower than that for ULTEM™ 9085, as evident in Figure 7. Only the Type 4 geometry exhibited failures, with an in-gage consistency greater than 70%.

A summary of the responses for the ±45° and 0/90° infill configurations is provided in Table 4. Notably, there were no acceptable failures within the narrow gage section for the 0/90° Dual Flange samples. In fact, few acceptable gage section failures were received from the 0/90° samples overall as defined by ASTM D638.

### 3.3. Failure Locations

**Figure 8:**
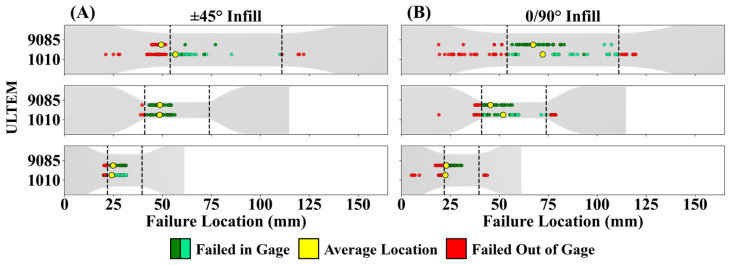
Failure locations within the ULTEM™ 9085 and 1010 coupons for the (**A**) ±45° infill and the (**B**) 0°/90° infill. Failures that do not conform to the ASTM standard are marked in red, and acceptable failures are marked in green/light green. Light green samples had multiple failures along the sample, with one in gage. The average overall failure location is indicated in yellow.

Figure 8 shows the location of failure for all sample configurations. The vertical dashed lines indicate the boundaries of the narrow section of the gage in which acceptable failures occur. Averaging across all sample configurations, only 60.5% of coupons tested failed within the region acceptable as defined by the standard.

**Figure 9:**
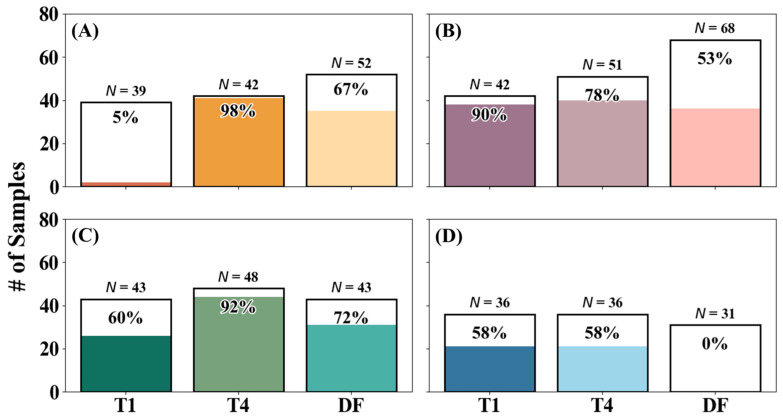
Bar charts showing the number of samples tested overlayed with the percentage of samples tested that failed within the ASTM acceptable region for each geometry. Results are shown for ±45° and 0/90° ULTEM™ 9085 in (**A**,**B**), respectively, and for ULTEM™ 1010 in (**C**,**D**), respectively. The total number of samples tested is presented at the top of each column (*N*).

There were samples of each geometry that failed within the region between the grip and gage section, hereafter referred to as the transition region. The exact location of these failures varied with material and sample geometry. In the case of the ULTEM™ 1010 Type 1 samples with ±45° infill, many failures involved multiple fracture sites, including within the gage section and the transition region. Due to some fractures occurring outside of the gage section, these responses could be categorized as invalid. However, they are considered acceptable here as long as one failure occurred in the gage section, as it is suspected the other failures are secondary and occur after the in-gage failure. Figure 9 summarizes the distribution of compliant failures across the different combinations of material and infill, based on Table 3 and Table 4. The results for the ±45° and 0/90 infills for ULTEM™ 9085 are shown in Figure 9A and Figure 9B, and those for 1010 are shown in Figure 9C and Figure 9D, respectively. The percentage of failures deemed acceptable under the standard is annotated for clarity. A stricter interpretation of the ASTM failure criteria is included in Appendix A, where only a single in-gage failure is deemed acceptable.

As evident from the presentation in Figure 9, the number of gage section failures is highly dependent on sample geometry and infill. According to the standard, *N* = 5 samples must break within the gage section for a valid UTS. However, for some infill and geometry combinations, few or no samples broke within the gage section. Namely, the Type 1 ULTEM™ 9085 ±45° samples and the Dual Flange ULTEM™ 1010 0/90° samples exhibited fewer than *N* = 5 failures within the gage section.

According to the results presented in Figure 8 and Figure 9, the UTS of the ULTEM™ 9085 with ±45° infill could only be qualified using the Type 4 and Dual Flange coupons; these two configurations resulted in *N* ≥ 5 gage section failures. And for ULTEM™ 9085 with 0/90° infill, a valid UTS was successfully obtained for all sample geometries. For ULTEM™ 1010 with ±45° and 0/90° infill, a valid UTS was successfully obtained for all sample geometries except for the 0/90° Dual Flange samples. For this sample and infill, no samples failed within the gage section.

## 4. Discussion

According to the elastic modulus of ULTEM™ 9085, ASTM D638 advises that the tensile properties should be evaluated using the T1 sample configuration [7,14,15]. However, the results of the experimental evaluation showed that for the ±45° infill, it is nearly impossible to achieve the required number (*N* = 5) of in-gage section failures with the T1 configuration. With a 5% “in-gage” failure rate (Figure 9), approximately 100 samples would be needed to qualify this material according to the standard. The results in Table 3 demonstrate that the location of failure cannot be ignored as the UTS is reduced, regardless of sample geometry, when all samples are included. While greater success was achieved overall for the 0/90° infill in evaluating ULTEM™ 9085, twice the required number of coupons was required to achieve *N* = 5 failures within gage section for the Dual Flange geometry. Similar trends related to failures outside the gage section have been reported in the literature. For instance, an average of only 63% failures occurred in the gauge section in a reported evaluation of ABS polymers using the Type 1 configuration [34]. Interestingly, for the ±45° infill, the T4 sample geometry was successful for both polymers in the present study and yielded comparable success for the 0/90° infill. However, according to ASTM D638, the T4 geometry is recommended for more compliant polymers and not for materials like ULTEM™. Surprisingly, while the newly developed Dual Flange geometry was successful for ULTEM™ 9085 regardless of infill, it failed to achieve a qualifiable UTS for the 0/90° infill of ULTEM™ 1010. None of the coupons failed within the gage section. This reduces the potential universality of the success of Dual Flange sample geometries in characterizing AM polymers.

A change is clearly needed to increase the consistency of failures in the gage section of ASTM samples and simplify the qualification process of AM materials. This change is a safeguard to prevent reporting properties that do not conform to the ASTM standard. It is especially important for accelerated aging studies. Most reported studies on the degradation of polymer materials have used the XY configuration to establish the baseline material properties of printed samples (e.g., [10,35,36]). Based on the findings presented here, these mesostructural defects that arise when using the default print parameters could misinform the effects of the degradation environment on these polymer systems. It is essential to determine whether the out-of-gage failures (Figure 8) and lower UTS results are due to morphological defects and sample topology that concentrate stress in the transition zone, or to other factors, such as the encapsulated porosity revealed by the CT scans in Figure 5.

What is the root cause of the out-of-gage section failures? Potential contributions include surface stress concentrations posed by the printed surface texture (Figure 4), internal voids within the rasters and/or voids at the interface of the rasters, as shown in Figure 3 and Figure 5. If surface defects were the dominant contributor to failure, similar failures would be expected across all sample geometries and within the gage length, where the nominal stress is the maximum. However, a large percentage of failures occurred within the gage-to-grip transition region, as seen in Figure 8. The measurements of surface roughness, particularly the R_v_ parameter, conveyed the degree of fusion between neighboring contours. Lower R_v_ values indicate improved inter-contour fusion, as the valleys between adjacent contours are reduced. As illustrated in Table 1 and Table 2, all samples exhibited R_v_ values greater than 50 µm, indicating that the depth between contours was over 10% of the individual contour thickness. As nearly 40% of all failures occurred within the transition region, the surface condition alone does not explain the strong localization of out-of-gage failures.

Internal porosity reduces tensile strength [37]. While the printed sample types may exhibit similar levels of porosity overall, variations in performance could be due to sporadic voids or geometry rather than porosity alone [38]. Figure 5 shows that the porosity measurements encompass a range of void sizes, including the printed mesostructure and voids within the infill rasters and contours, as presented in Figure 5C. While small encapsulated microstructural voids were occasionally seen in fracture surfaces, larger interconnected open pores were evident throughout the printed body of the samples. Since the largest nominal stress develops in the gage section, distributed encapsulated microstructural voids cannot be the root cause of failure.

The localization of failures outside the gage section appears to result predominately from the as-printed mesostructure generated by the default processing parameters. Stress concentrations arise from the transition region mesostructure and are associated with the reduction in cross-section and corresponding raster design that causes incomplete filling. These regions coincide with the locations of failure identified in Figure 8 and the interconnected open pores that exist throughout the body of the samples, as seen in Figure 3. A comparative assessment of the different coupon architectures suggests that modifying the raster–raster overlap could mitigate these stress concentrations [39,40]. A modification to this raster–raster overlap promotes mechanistic improvements such as the diffusion, entanglement, and healing of the polymer chains between adjacent printed contours. All three of these mechanisms work to improve the printed microstructure, facilitate polymer chain diffusion across interfaces and reduce the presence of internal notch effects posed by stress concentration [41]. This adjustment can be implemented without altering the underlying mesostructure and strengths in the XZ and ZX build orientations. The ZX orientation is largely governed by inter-laminar strength, whereas the XZ orientation relies on the strength of wall contours. Implementing these changes should enable a more accurate evaluation of the mechanical properties of ULTEM™ in the printed condition, independent of mesostructural artifacts.

### 4.1. Weibull Analysis

**Figure 10:**
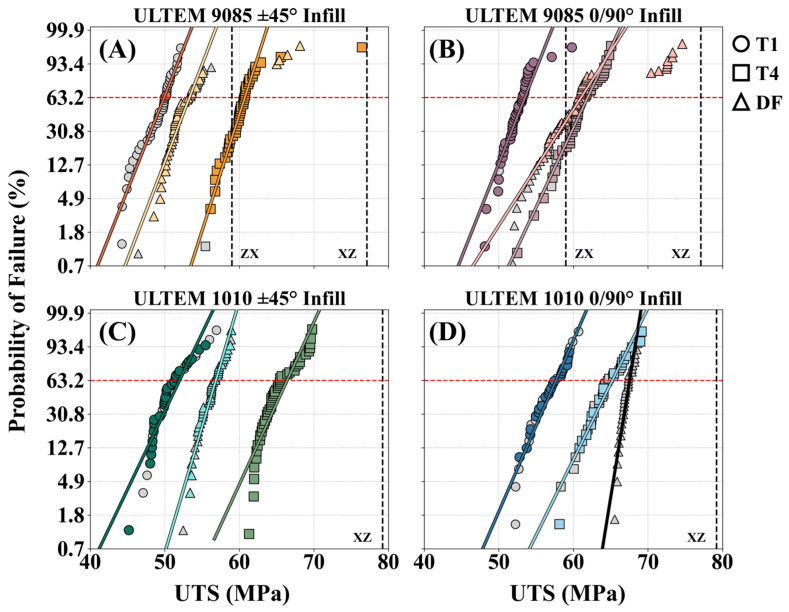
Weibull distributions for the UTS of the ULTEM™ 9085 and 1010 materials. (**A**) ULTEM™ 9085, ±45°infill; (**B**) ULTEM™ 9085, 0/90° infill; (**C**) ULTEM™ 1010, ±45° infill; and (**D**) ULTEM™ 1010, 0/90° infill. The vertical black dashed lines represent the Stratasys ZX and XZ strengths for ULTEM™ 9085 (59 MPa, 77.1 MPa) and 1010 (28.2 MPa, 79.2 MPa). Colored markers failed in-gage.

The UTS measurements were evaluated using a two-parameter Weibull distribution and are presented in Figure 10 regardless of failure location. Specifically, the results for the ±45° and 0/90° infills are shown for ULTEM™ 9085 in Figure 10A and Figure 10B, respectively, and for ULTEM™ 1010 in Figure 10C and Figure 10D, respectively. The data is coded to indicate failures that occurred within (color) and outside (gray) the gage section. The values provided in the material datasheet by Stratasys for the ZX and XZ (on-edge) configurations are shown in these figures as vertically dashed black lines. In addition, a horizontal dashed red line is included at 63.2% probability, defining the characteristic strength (σ_0_) [14,15]. The Weibull modulus (m) defines the slope of the data, which reflects reliability. In general, the data for each sample geometry follows a straight line, suggesting that the Weibull model is acceptable. The results presented in Figure 10A,B for the Dual Flange samples exhibit two distinct groups, suggesting multiple failure modes or root causes. The smaller group of “high-performing” samples was excluded from the calculation of m and σ_0_ for the Dual Flange samples. These are discussed separately alongside the two outliers from the ±45° group of Type 4 ULTEM™ 9085.

The characteristic strength of the Type 4 samples (Figure 10) is consistently the highest in all cases, except for ULTEM™ 1010 with 0/90° infill, where the Dual Flange strength is the highest. Surprisingly, none of these Dual Flange coupons failed within the gage section. The combination of high Weibull modulus and unacceptable failures indicates that the mesostructure at the end of the transition zone is the weakest link in this coupon geometry and contains a defect that consistently serves as the origin and root cause of failure. The Type 1 coupons consistently displayed the lowest characteristic strength in comparison to the results for the Type 4 geometry. The results from Figure 10 are summarized in Table 5.

The UTS distribution for the Dual Flange samples in Figure 10A,B exhibits two distinct groups, suggesting unique failure modes. Based on fractography, the samples with performance exhibited a substantially larger degree of inter-contour bonding, approaching 25% of the total cross-sectional area. In contrast, the low-performing coupons exhibited approximately 5% inter-contour bonding within the cross-section. In addition, voids were visible within the contours on the fracture surface. Additional images and details are available in the Appendix A. For ULTEM™ 1010, which exhibited a larger number of failures outside the gage section overall, the 0/90° infill groups exhibited the poorest performance, with only half of the Type 1 and Type 4 samples failing in the gage section. For the 0/90° Dual Flange samples, all failed at the end of the transition region, just before the gage section. For these coupons, a large void was observed within the transition zone at the contour raster intersection due to the default slicing parameters used with this geometry. The consistency in this structural defect resulted in failures that always initiated at this location, and thus led to a high Weibull modulus, but notably no failures that conformed to the standard. This void and the lower strain to failure of ULTEM™ 1010 were the primary contributions to the behavior observed in Figure 10 [42].

The Weibull distributions, combined with the results of fractography, suggest that a change in microstructure is necessary to overcome problems with qualification. Other investigators who have adopted ASTM D638 for evaluating thermoplastics and identified issues have adopted alternative standards, such as those for composite materials (ASTM D3039) or by making selective improvements to the mesostructure of the coupons [43,44]. It would be ideal if all three sample types yielded the same mechanical property estimates, thereby reflecting the material rather than the sample geometry. This is particularly true for the XY (flat) configuration, where the printed microstructure appears more problematic than in the XZ and ZX configurations. In the flat configuration, changes to the geometry affect the number of raster contour intersections in printing both the ±45° and 0/90° infill coupons. Hence, it may be possible to overcome these defects at intersections and related contour-to-contour interactions through modifications to the printing contour design.

### 4.2. Improving the Printed Mesostructure

The optical evaluation of the printed mesostructure revealed voids at the contour and raster intersections within all three sample types, as well as both infill configurations. These voids caused out-of-gage section failures that invalidated some of the mechanical property assessments. It is possible to adjust the airgap parameter in Insight to refine the microstructure and reduce the severity of these features that served as the root cause of failure. Specifically, the overlap between the deposited elements can be tuned by adjusting the airgap parameter to minimize these voids [45]. In addition, the raster and contour widths can be reduced to enable more nozzle passes and a greater infill density, which reduces the size of contour intersection voids.

**Figure 11:**
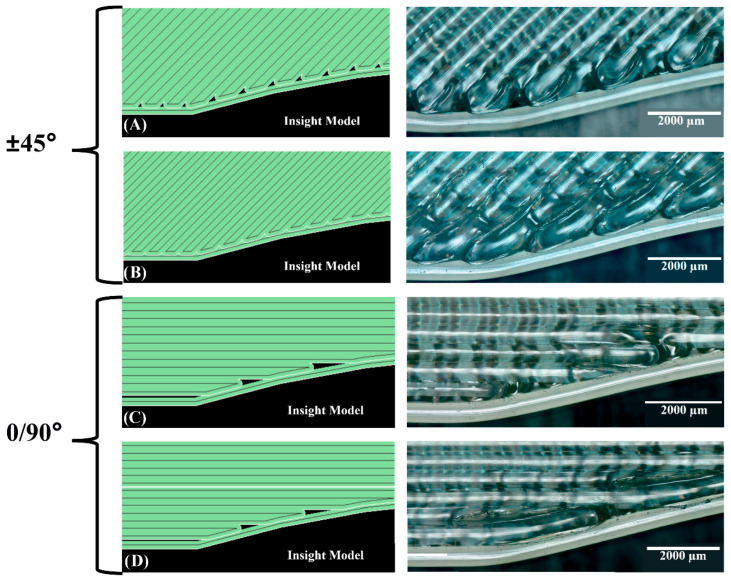
The transition region of ASTM Type 1 samples printed with ULTEM™ 9085 in the XY plane. The printing results are shown for the default and 0.0254 mm overlap conditions with ±45° infill in (**A**,**B**) and for the 0/90° infill in (**C**,**D**), respectively.

Additional samples of ULTEM™ 9085 were printed with modified infill and overlap definition. While modifying the raster and contour width parameters increased infill density, UTS did not increase, consistent with the results of Gabisa et al. [46]. Regarding overlap, additional Type 1 samples of ULTEM™ 9085 were printed with overlaps of 0.0, 0.025, 0.031, and 0.038 mm. Overall, the samples printed with a negative airgap (i.e., with an overlap) showed improvements in microstructure and strength. However, overlaps ≥0.030 mm resulted in polymer buildup on the nozzle orifice, which caused defects within and on the surface of the samples. The samples printed with an overlap of 0.0254 mm were optimal and did not exhibit these defects. A comparison of the microstructure resulting from the default parameters and with overlap is shown in Figure 11.

**Figure 12:**
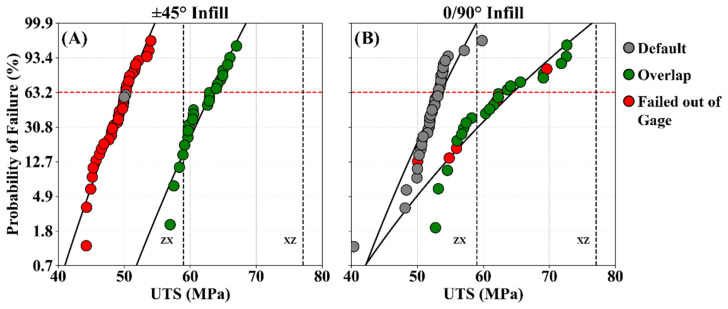
Weibull distributions for the Type 1 samples prepared with default and overlap (−0.0254 mm) conditions for the (**A**) ±45° and (**B**) 0/90° infill print patterns. Note that the responses include the results of all samples tested, including those that failed outside and within the gage sections.

A comparison of the Weibull distributions for the UTS of samples with the default print settings and 0.0254 mm overlap is shown in Figure 12. The default and modified infill responses are shown for the ±45° and 0/90° infill samples in Figure 12A and Figure 12B, respectively. The Weibull modulus and characteristic strength of the ±45° infill samples with overlap were 31.1 and 61.7 MPa, and for the 0/90° infill, these failures were 11.7 and 66.0 MPa, respectively. The modified infill resulted in a 23% improvement in characteristic strength relative to the default printing conditions. Although, the Weibull modulus increased for the ±45° infill, it decreased for the 0/90° infill samples, which is indicative of a change in the failure mode.

In addition to UTS, the results in Figure 12 also identify specimens that failed outside of the gage section. Clearly, increasing the overlap from the default 0 mm to 0.0254 mm increased the percentage of failures in the gage section. For the ±45° infill, gage section failures increased from roughly 5% to 100%. For the 0/90° infill samples, it remained at approximately 80%. Correspondingly, the UTS of these samples increased from 49 ± 2 MPa to 61 ± 2 MPa and from 53 ± 3 MPa to 63 ± 6 MPa, respectively. These corrected mesostructures now have mechanical responses that align with the highest reported values for AM ULTEM™ in the literature (65 MPa) [18]. These results underscore the important role of the overlap region in removing transition region voids and in qualifying AM materials for prints in the XY plane. The same benefits should be realized in component performance.

**Figure 13:**
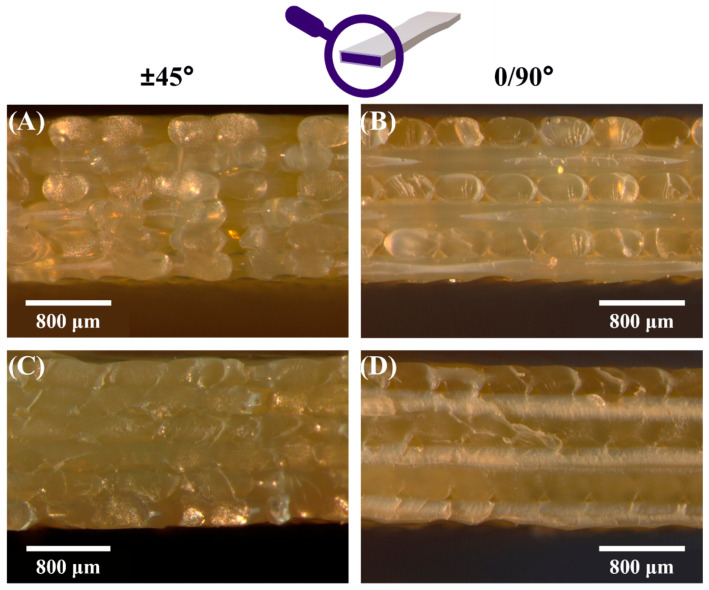
The fracture surfaces of ULTEM™ 9085. (**A**,**B**) Default printing parameters and (**C**,**D**) the −0.0254 mm overlap for the 0/90° and ±45° infill. Note the increase in raster interface bonding that is evident, especially for the 90° rasters in (**D**).

Representative facture surfaces of a Type I coupon of ULTEM™ 9085 with ±45° infill are shown for the default print settings and overlap in Figure 13A and Figure 13C, respectively. Similarly, for the 0/90° infill, representative fracture surfaces are shown in Figure 13B and Figure 13D, respectively.

There was a notable improvement in fusion at the raster interfaces and with the exterior contours surrounding the perimeter of the coupons (Figure 13). These observations highlight the substantial importance of process control on the mesostructure of components produced by FDM and its effect on strength and reliability. Samples printed with a modified overlap exceed the values reported for the ZX configuration, which shows that the modified XY structure is stronger than that of interlayer adhesion alone. It is notable that interlayer adhesion strength is not achieved under the default printing conditions, as shown in Figure 10. Furthermore, the strength is approaching that of the XZ orientation. Nevertheless, there are additional features that limit the UTS from reaching that of the XZ orientation in the Stratasys datasheet for ULTEM™ 9085 [14].

Although the characteristic strength improved with an overlap of 0.0254 mm for both the ±45° and 0/90° infill samples, the UTS distribution of the modified coupons exhibited a lower Weibull modulus (Figure 12). The improved fusion between the raster and contour of the samples improved load transfer in the transition region, causing failures to initiate at the weakest links in the gage section of the sample.

The results obtained with the improved overlap definition and the corresponding mesostructure in Figure 11, Figure 12 and Figure 13 show that Type 1 ASTM D638 samples can be used reliably to qualify the mechanical properties of engineering thermoplastics printed by FDM. However, the proper implementation of the overlap parameters is essential for ensuring contour fusion and maximizing printed strength. Adjusting the overlap parameter increased the sample failure rate within the gage section, simplifying the qualification process for these materials and reducing the total number of samples required for testing. Based on the findings presented in this study, in the near term, it is recommended that AM users consider implementing material overlap adjustments like those presented here, to assist in material qualification and yield mechanical properties reflective of the material system rather than the printed mesostructure. Considerations regarding the long-term suitability of ASTM D638 for the qualification of AM polymers are needed. Significant benefits could be realized through the development of an AM-specific material qualification framework, grounded in rigorous statistical evaluation and designed to minimize the reported range of anisotropic properties in printed specimens. Reported studies in this space have rarely shared the results of fractography. Given the important relationship between mechanical properties and fracture origin identified herein, it is recommended that future studies explicitly include these metrics.

### 4.3. Limitations

The findings of this study provide new understanding concerning the challenges encountered in applying ASTM D638 to characterize the mechanical properties of ULTEM™ 9085 and 1010 and distinguish a route for: (1) qualifying prints with XY orientation and (2) improving the strength and reliability of printed components. However, there are limitations to the investigation that should be considered. The samples printed with the modified overlap parameter were produced on a Fortus 400mc. This system is incompatible with the T14A and T16A tips used on the Fortus 900mc to print the default Type 1, Type 4, and Dual Flange samples. It is possible that higher strength and reliability could be achieved with the utilization of these aerospace-grade tips. Additional performance gains may be achieved by using Certified Grade ULTEM™, which is regarded for its traceability and higher performance compared to the Standard Grade. Nevertheless, the benefits of the overlap definition were not obscured by this issue. Furthermore, an evaluation of the feedstock material, independent of the effects of printed mesostructures, could support a more rigorous determination of material strength before evaluating printed structures. For example, polymer strength could be assessed from an evaluation of the individual filaments or contours of the printed material to establish baseline material properties. Another limitation is that the investigation was focused on ULTEM™ materials and did not include other high-stiffness AM polymers, e.g., PEEK, PEK, etc. Future work should consider changing the overlap parameter from the default during the qualification of other FDM polymers to establish material properties relative to the MDS in the XZ, ZX, and XY planes. Overall, the investigators hope that the results of this investigation are helpful in the qualification of AM polymers.

## 5. Conclusions

Based on the experimental results, the following conclusions were drawn:

1. The UTS of ULTEM™ 9085 and 1010 determined using the Type 1, Type 4, and Dual Flange coupons was highly dependent on the unique failure modes of the printed samples. For the recommended printer settings, the ±45° infill configuration could not be qualified with the Type 1 sample geometry for ULTEM™ 9085. Similarly, the 0/90° infill configuration could not be qualified with the Dual Flange sample geometry for ULTEM™ 1010.
2. The Type 4 sample geometry resulted in the largest number of in-gage section failures overall, with 41/42 (97%) and 41/50 (82%), respectively, for the ±45° and 0°/90° infill orientations. Out-of-gage section failures initiated at voids in the transition region at the raster/contour interfaces.
3. Each sample geometry exhibited distinct locations of failure according to the infill pattern and location of these defects. However, the root cause of the out-of-gage section failures was voids along the contour–raster interface, which created stress concentrations.
4. Modifications to the raster pattern were introduced using an overlap definition to reduce the presence of voids along the contour–raster interface. With an optimum overlap of 0.0254 mm, the UTS of the Type 1 ±45° samples increased from 49 ± 2 MPa to 61 ± 2 MPa (24% increase). For the 0/90° samples, the UTS increased from 53 ± 3 MPa to 63 ± 6 MPa (19% increase). These modifications markedly decreased anisotropy in the printed material, resulting in statistically insignificant variation in UTS with respect to infill pattern or build orientation.
5. The modified overlap also resulted in a significantly larger percentage of in-gage section failures, resulting in reduced time to qualification. For the ULTEM™ 9085 Type 1 samples printed with ±45° infill, the percentage of within-gage section failures improved from 5% to 100%. For the 0/90° infill orientation, the percentage reached approximately 80%.

## Figures and Tables

**Table 1:** Surface roughness measurements of ULTEM™ 9085 samples.

ULTEM™ 9085 Sample Type	Top Face			Build Plate		
	R_a_ (µm)	R_v_ (µm)	R_vk_ (µm)	R_a_ (µm)	R_v_ (µm)	R_vk_ (µm)
T1, ±45°	17.0	73.8	64.8	18.6	75.3	73.0
T4, ±45°	13.2	51.9	54.4	27.1	93.7	90.0
DF, ±45°	16.5	67.3	64.0	34.4	112.0	130.8
T1, 0/90°	26.0	105.4	83.6	16.4	87.1	80.3
T4, 0/90°	21.9	91.8	70.6	26.1	103.4	92.2
DF, 0/90°	22.6	87.4	74.2	35.0	120.4	114.6

**Table 2:** Surface roughness measurements of ULTEM™ 1010 samples.

ULTEM™ 1010 Sample Type	Top Face			Build Plate		
	R_a_ (µm)	R_v_ (µm)	R_vk_ (µm)	R_a_ (µm)	R_v_ (µm)	R_vk_ (µm)
T1, ±45°	19.6	78.7	73.6	24.3	88.9	66.8
T4, ±45°	19.8	82.4	77.5	26.9	84.2	66.3
DF, ±45°	20.2	81.8	78.0	15.3	72.6	66.9
T1, 0/90°	30.9	121.9	99.3	23.1	99.0	61.4
T4, 0/90°	22.5	83.5	70.2	24.7	93.7	74.7
DF, 0/90°	23.8	90.3	75.4	24.6	88.3	72.3

**Table 3:** Mechanical properties for ULTEM™ 9085 samples obtained in accordance with ASTM D638.

Sample Type	*N* Tested	In-Gage Failure	UTS (in Gage) ± Std. Dev. (MPa)	UTS (All Samples) ± Std. Dev. (MPa)
T1, ±45°	39	2	50.1 ± 0.1	48.9 ± 2.2
T4, ±45°	42	41	60.4 ± 3.1	60.3 ± 3.2
DF, ±45°	52	35	53.2 ± 5.0	53.2 ± 4.8
T1, 0/90°	42	38	52.5 ± 2.1	52.5 ± 2.6
T4, 0/90°	51	40	60.8 ± 2.8	60.7 ± 2.6
DF, 0/90°	68	36	63.0 ± 5.9	60.4 ± 3.2

**Table 4:** Mechanical properties for ULTEM™ 1010 samples obtained in accordance with ASTM D638.

Sample Type	*N* Tested	In-Gage Failure	UTS (in Gage) ± Std. Dev. (MPa)	UTS (All Samples) ± Std. Dev. (MPa)
T1, ±45°	43	26	50.5 ± 2.3	50.8 ± 2.5
T4, ±45°	48	44	65.2 ± 2.4	65.2 ± 2.4
DF, ±45°	43	31	56.3 ± 1.5	56.1 ± 1.6
T1, 0/90°	36	21	56.7 ± 2.0	56.5 ± 2.4
T4, 0/90°	36	21	64.4 ± 2.8	64.0 ± 2.7
DF, 0/90°	31	0	N/A ± N/A	67.1 ± 0.9

**Table 5:** Weibull parameters for ULTEM™ 9085 and 1010.

**ULTEM™ 9085**	**T1, ±45°**	**T4, ±45°**	**DF, ±45°**	**T1, 0/90°**	**T4, 0/90°**	**DF, 0/90°**
Modulus	26.9	40.9	30.2	30.2	28.3	20.6
σ_0_ (MPa)	49.9	60.7	53.3	53.5	61.9	61.0
**ULTEM™ 1010**	**T1, ±45°**	**T4, ±45°**	**DF, ±45°**	**T1, 0/90°**	**T4, 0/90°**	**DF, 0/90°**
Modulus	24.0	31.9	42.0	28.7	28.8	92.3
σ_0_ (MPa)	51.9	66.4	56.8	57.6	65.2	67.5

**Figure A1:**
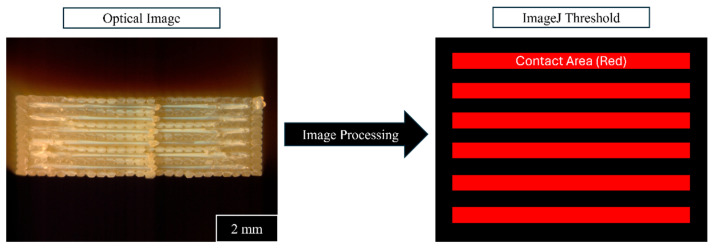
Fracture surface of ULTEM™ 9085, 0/90° infill DF sample showing idealized view of contact area between adjacent printed contours.

**Figure A2:**
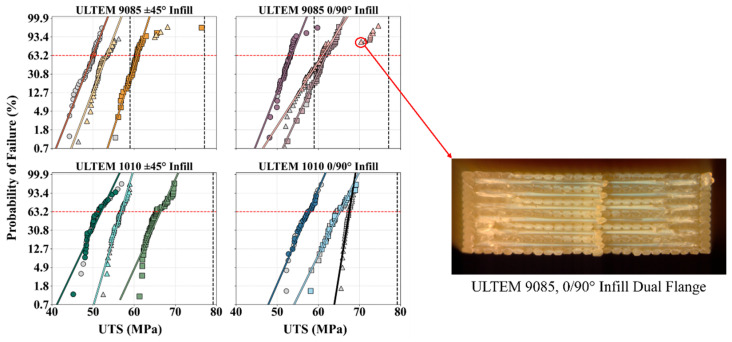
Fracture surface of ULTEM™ 9085, 0/90° infill (high-performing) DF sample with exploded view from its position in Weibull distribution. Colored markers failed in-gage.

**Figure A3:**
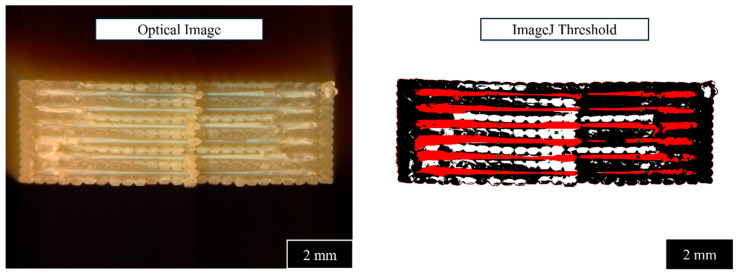
Fracture surface of ULTEM™ 9085, 0/90° infill (high-performing) DF sample, ImageJ, showing 24% contact between contours across total surface area measured.

**Figure A4:**
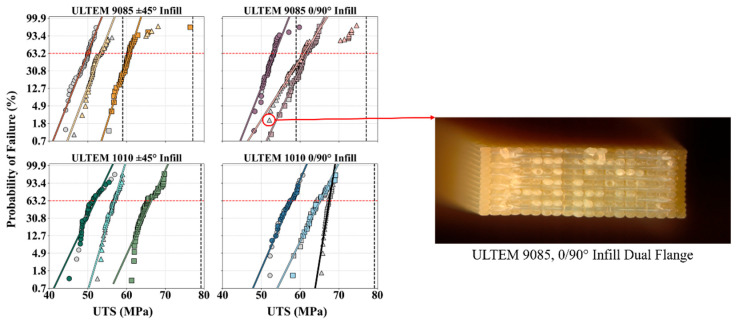
Fracture surface of ULTEM™ 9085, 0/90° infill (low-performing) DF sample with exploded view from its position in Weibull distribution. Colored markers failed in-gage.

**Figure A5:**
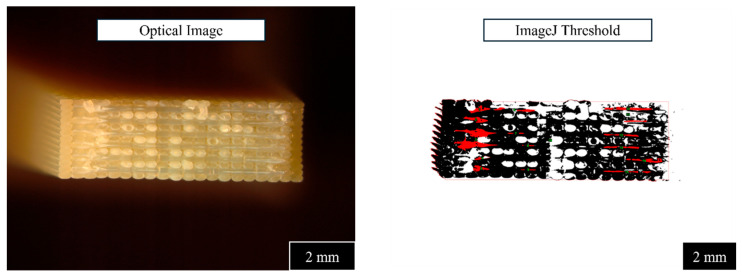
Fracture surface of ULTEM™ 9085, 0/90° infill (low-performing) DF sample, ImageJ, showing 5% contact between contours across total surface area measured.

**Figure A6:**
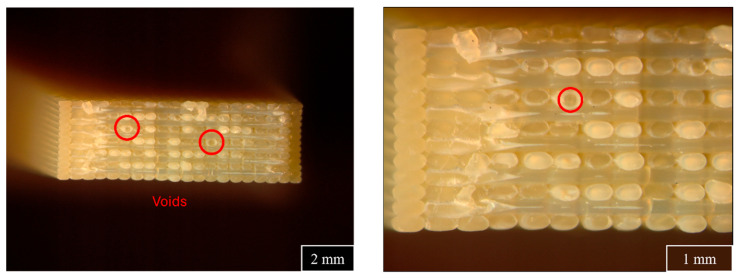
Fracture surface of ULTEM™ 9085, 0/90° infill (low-performing) DF sample at low and high magnification, highlighting void defects present within sample.

**Figure A7:**
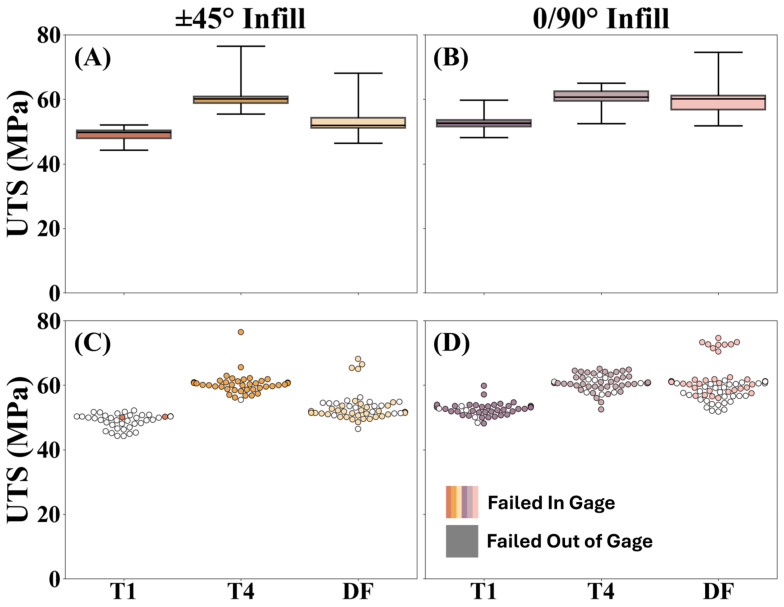
UTS values for ULTEM™ 9085 samples with a single failure in-gage and with ±45° (**A**,**C**) and 0/90° (**B**,**D**) raster patterns showing statistical distribution in strength (box and whisker (**A**,**B**)) and individual sample failure location with strength (swarm (**C**,**D**)).

**Figure A8:**
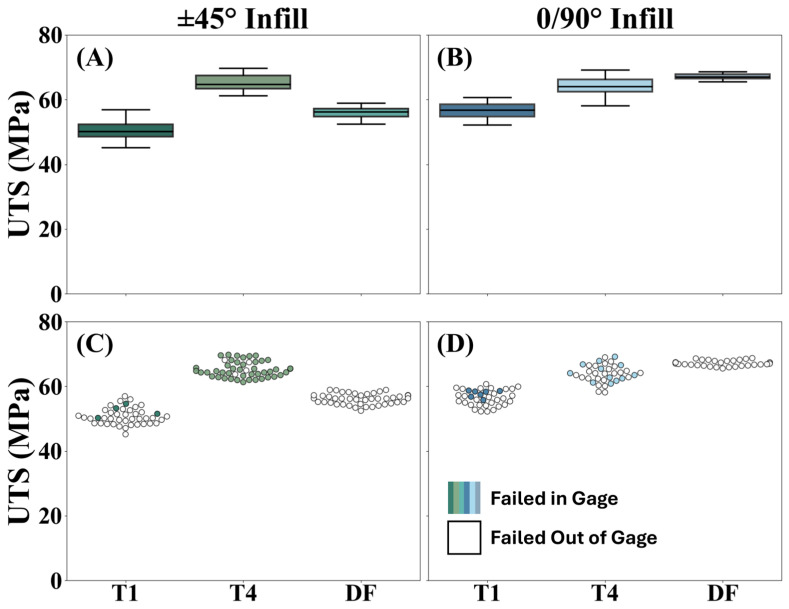
UTS values for ULTEM™ 1010 sample with a single failure in-gage and with ±45° (**A**,**C**) and 0/90° (**B**,**D**) raster patterns showing statistical distribution in strength (box and whisker (**A**,**B**)) and individual sample failure location with strength (swarm (**C**,**D**)).

**Figure A9:**
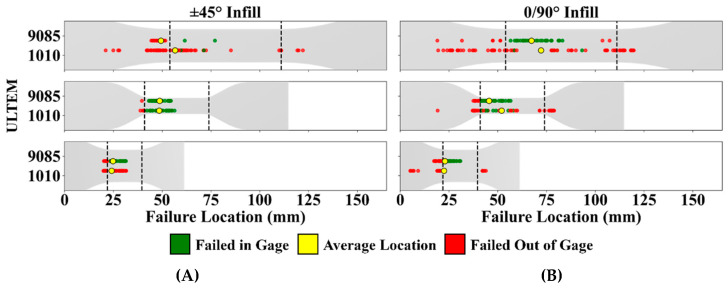
Failure locations within the ULTEM™ 9085 and 1010 coupons for the (**A**) ±45° infill and the (**B**) 0°/90° infill. Failures that do not conform to the ASTM standard are marked in red, and acceptable failures are marked in green. The average overall failure location is indicated in yellow.

**Figure A10:**
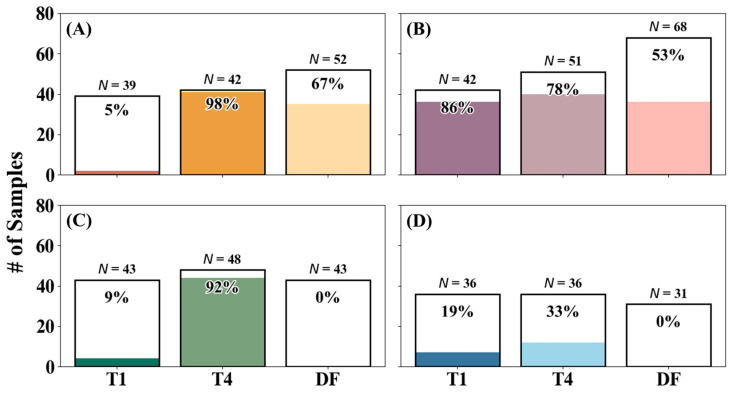
Bar charts showing the number of samples tested overlayed with the percentage of samples tested that failed within the ASTM acceptable region for each geometry. Results are shown for ±45° and 0/90° ULTEM™ 9085 in (**A**,**B**), respectively, and for ULTEM™ 1010 in (**C**,**D**), respectively. The total number of samples tested is presented at the top of each column (*N*).

**Figure A11:**
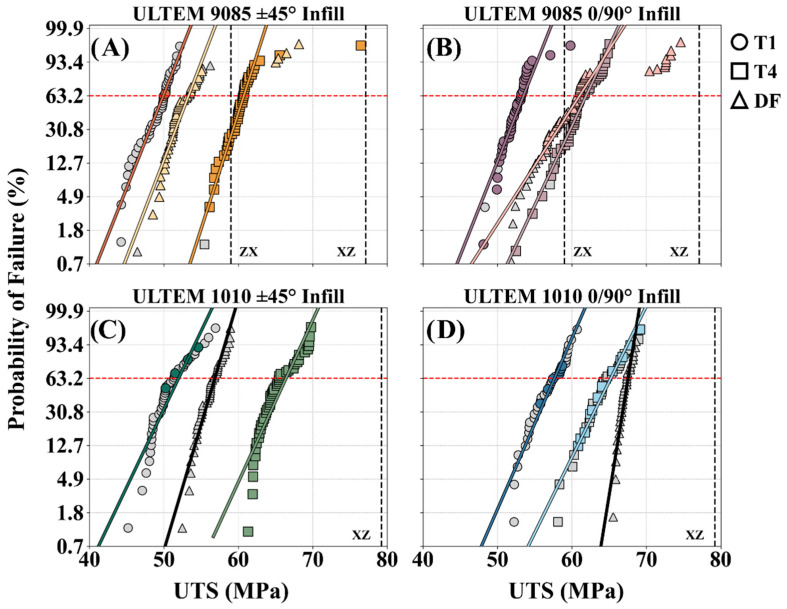
Weibull distributions for the UTS of the ULTEM™ 9085 and 1010 materials with a single in-gage failure. (**A**) ULTEM™ 9085, ±45°infill; (**B**) ULTEM™ 9085, 0/90° infill; (**C**) ULTEM™ 1010, ±45° infill; and (**D**) ULTEM™ 1010, 0/90° infill. The vertical black dashed lines represent the Stratasys ZX and XZ strengths for ULTEM™ 9085 (59 MPa, 77.1 MPa) and 1010 (28.2 MPa, 79.2 MPa). Colored markers failed in-gage.

**Figure A12:**
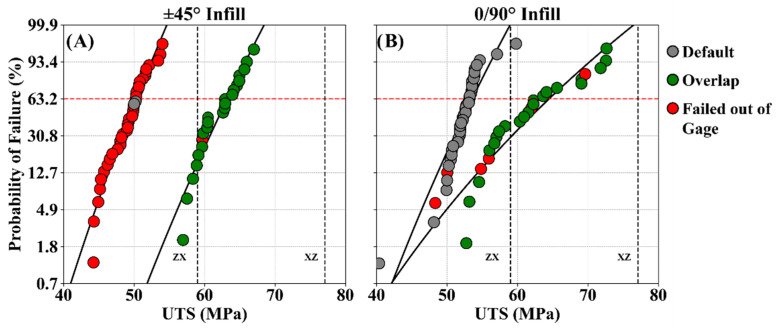
Weibull distributions for the Type 1 samples prepared with default and overlap (−0.0254 mm) conditions for the (**A**) ±45° and (**B**) 0/90° infill print patterns. Note that the responses include the results of all samples tested, including those that failed outside and within the gage sections.

## Data Availability

The raw data supporting the conclusions of this article will be made available by the authors on request.

## Abbreviations

The following abbreviations are used in this manuscript:

AM	Additive Manufacturing
CAD	Computer-Aided Design
FDM	Fused Deposition Modeling
FFF	Fused Filament Fabrication
MDS	Material Datasheet
Micro-CT	Micro-Computed Tomography
PC	Polycarbonate
PEI	Polyetherimide
UTS	Ultimate Tensile Strength
UUVs	Unmanned Underwater Vehicles

## Appendix A

### Appendix A.1

Two distinct sample groupings can be identified in Dual Flange samples printed with 0/90° infill, and the following images presented in Appendix A.1 outline the process that was used to determine that this grouping is likely due to the increased contact area between printed contours in a small subset of printed samples. It is hypothesized that there is greater variation in contour fusion for these Dual Flange samples compared to either the Type 1 or Type 4 coupons, as the Dual Flange samples require a greater amount of support material underneath when laid flat on the XY plane in Insight. Evaluations of optical fracture surfaces was performed in ImageJ, version 1.54p, using simple thresholding criteria to identify areas between contours where polymer chain entanglement occurred.

### Appendix A.2

The following figures present data where only a single failure within the gage section is deemed conforming by the ASTM D638. Notably, this stricter criteria for failure reclassified many failures for the default print condition samples but only a single sample for those printed with the overlap parameter modified.
